# Supplementary material for: Longitudinal, Intra-Individual Stability of Untargeted Plasma and Cerebrospinal Fluid Metabolites
Source: Metabolites. 2025 Dec 30;16(1):35. doi: 10.3390/metabo16010035 (PMC12844231; doi:10.3390/metabo16010035)
Supplement: Supplementary file 1 [file metabolites-16-00035-s001.zip › supplemental_figures_and_tables.pdf]

# Supplemental Figures and Tables

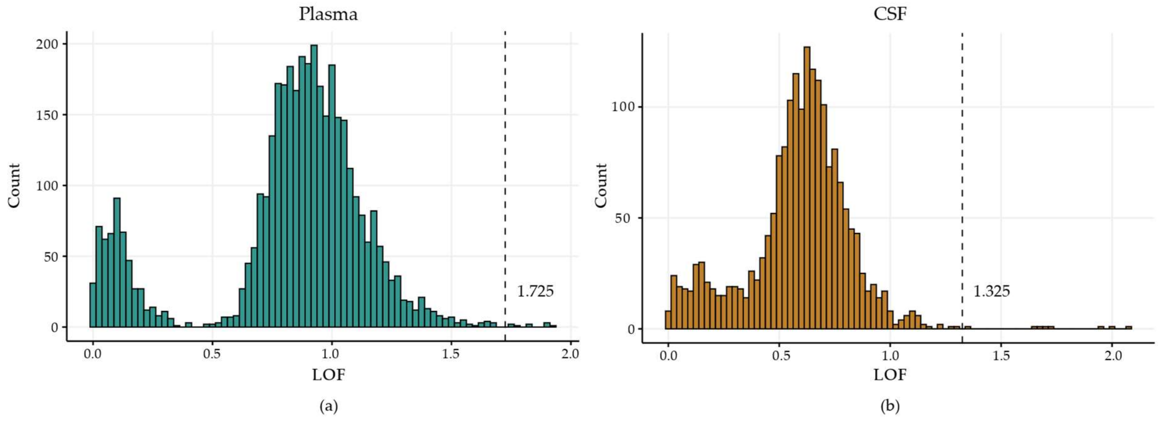


**Supplemental Figure S1.** Histograms of local outlier factors calculated for plasma (a) and CSF (b). Dashed lines represent the thresholds used to identify outliers. Samples with an LOF above each of these thresholds were removed (n = 9 participant samples from plasma; n = 8 samples from CSF including 4 pooled QC samples and 4 participant samples)

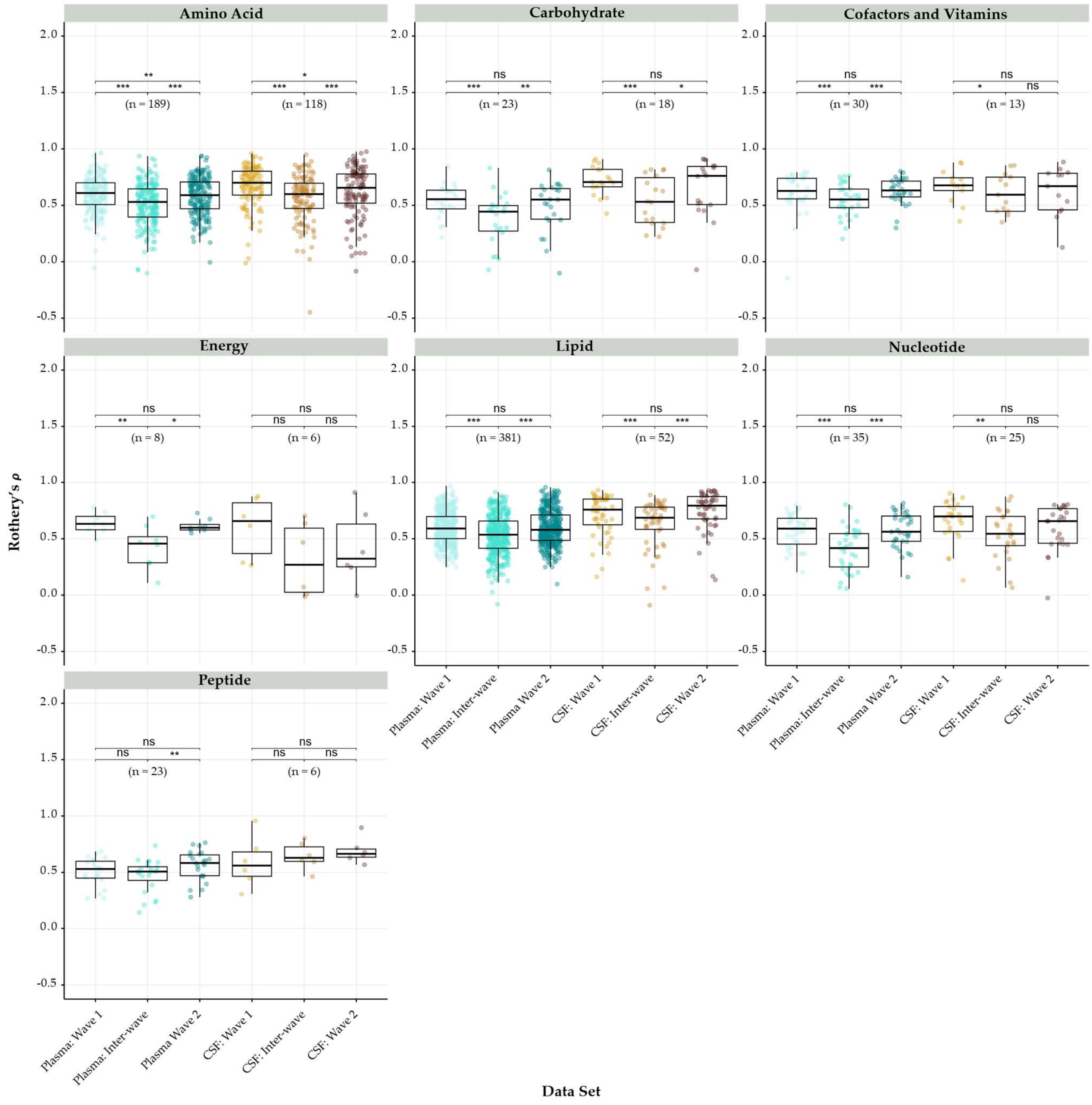


**Supplemental Figure S2.** Boxplots of Rothery's $\rho$ for metabolites by super pathway in plasma (teal) and CSF (tan). Each point represents an individual metabolite, with point color indicating data set (categorical). The n values correspond to the number of metabolites analyzed. The bold line within each box represents the median value for each data set. Asterisks indicate degree of significance for pairwise comparisons and were determined using p-values from Wilcoxon rank-sum tests (* = p <0.05, ** = p <0.01, *** = p<0.001, 'ns' = not significant).

**Supplemental Table S1.** Number of metabolites analyzed, median Rothery's $\rho$, and median $CV_{QC}$ values are displayed for each metabolite super pathway for plasma (top) and CSF (bottom). Interquartile ranges are displayed in parentheses. Xenobiotic, partially characterized and unnamed molecules were excluded from this analysis.

| | | **Plasma** | | | | | |
|---|---|---|---|---|---|---|---|
| | **n[1]** | **Median $\rho$ (IQR)** | | | **Median $CV_{QC}$ (IQR)** | | |
| **Super Pathway** | | **Wave 1** | **Inter-wave** | **Wave 2** | **Wave 1** | **Inter-wave** | **Wave 2** |
| Amino Acid | 189 | 0.61 (0.19) | 0.53 (0.25) | 0.59 (0.24) | 11.1 (8.7) | 11.5 (9.9) | 10.5 (10.8) |
| Carbohydrate | 23 | 0.55 (0.17) | 0.44 (0.23) | 0.55 (0.27) | 18.0 (18.3) | 15.8 (14.1) | 15.3 (13.9) |
| Cofactors and Vitamins | 30 | 0.63 (0.18) | 0.55 (0.16) | 0.63 (0.14) | 12.4 (7.4) | 12.4 (6.2) | 11.4 (5.8) |
| Energy | 8 | 0.63 (0.12) | 0.46 (0.23) | 0.60 (0.05) | 12.5 (14.9) | 12.4 (12.6) | 10.2 (14.3) |
| Lipid | 381 | 0.59 (0.2) | 0.54 (0.24) | 0.58 (0.23) | 12.3 (10.8) | 10.9 (9.1) | 9.0 (8.8) |
| Nucleotide | 35 | 0.59 (0.23) | 0.42 (0.3) | 0.56 (0.22) | 15.0 (6.8) | 13.9 (7.8) | 14.5 (9.3) |
| Peptide | 23 | 0.53 (0.15) | 0.51 (0.12) | 0.58 (0.19) | 11.8 (8.9) | 13.0 (5.9) | 10.0 (5.1) |
| | | **CSF** | | | | | |
| Amino Acid | 118 | 0.70 (0.21) | 0.60 (0.22) | 0.66 (0.26) | 11.0 (6.5) | 11.5 (5.7) | 11.4 (7.1) |
| Carbohydrate | 18 | 0.71 (0.15) | 0.53 (0.40) | 0.76 (0.34) | 11.0 (5.4) | 10.6 (4.5) | 10.1 (3.4) |
| Cofactors and Vitamins | 13 | 0.68 (0.11) | 0.59 (0.30) | 0.67 (0.32) | 12.3 (5.8) | 11.7 (12.3) | 9.5 (20.0) |
| Energy | 6 | 0.66 (0.45) | 0.27 (0.57) | 0.32 (0.38) | 12.0 (12.1) | 16.1 (14.1) | 14.9 (13.2) |
| Lipid | 52 | 0.76 (0.23) | 0.69 (0.20) | 0.79 (0.20) | 87.2 (264.7) | 74.4 (248.9) | 19.8 (30.8) |
| Nucleotide | 25 | 0.70 (0.22) | 0.54 (0.26) | 0.66 (0.31) | 10.7 (5.9) | 12.7 (4.6) | 11.2 (3.9) |
| Peptide | 6 | 0.56 (0.22) | 0.63 (0.13) | 0.66 (0.07) | 14.6 (8.7) | 13.1 (7.2) | 10.5 (3.6) |

[1]n corresponds to number of metabolites analyzed in each super pathway

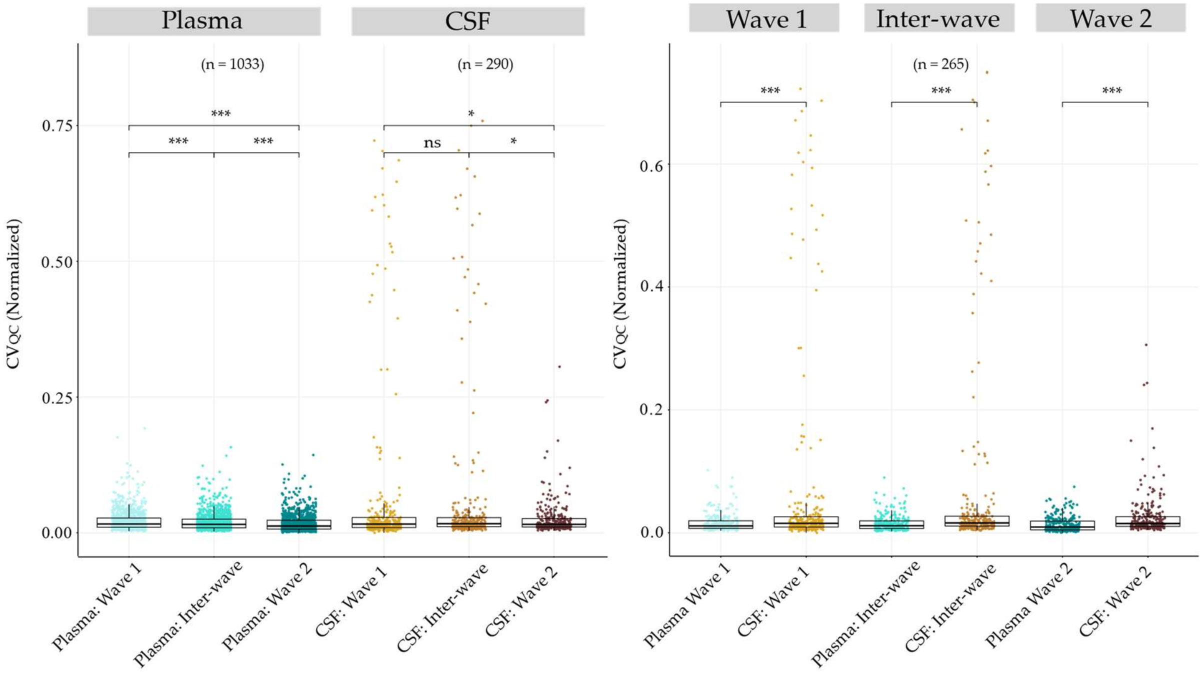


(a) (b)

**Supplemental Figure S3**. Boxplots of normalized CV measurements in pooled QC samples across all metabolites (a) and metabolites detected in both plasma and CSF (b). Brackets and asterisks represent the level of significance as determined by p-values calculated using paired Wilcoxon rank sum test (* = $p < 0.05$, ** = $p < 0.01$, *** = $p < 0.001$, 'ns' = not signifigant).
